# Sexual Dimorphism in Alcohol Induced Adipose Inflammation Relates to Liver Injury

**DOI:** 10.1371/journal.pone.0164225

**Published:** 2016-10-06

**Authors:** Melissa A. Fulham, Pranoti Mandrekar

**Affiliations:** 1 Department of Medicine, University of Massachusetts Medical School, Worcester, Massachusetts, United States of America; 2 Graduate School of Biomedical Sciences, University of Massachusetts Medical School, Worcester, Massachusetts, United States of America; University of Louisville School of Medicine, UNITED STATES

## Abstract

Alcoholic liver disease occurs due to chronic, heavy drinking and is driven both by metabolic alterations and immune cell activation. Women are at a higher risk than men for developing alcohol induced liver injury and this dimorphism is reflected in animal models of alcoholic liver disease. The importance of adipose tissue in alcoholic liver disease is emerging. Chronic alcohol consumption causes adipose tissue inflammation, which can influence liver injury. Sex differences in body fat composition are well known. However, it is still unclear if alcohol-induced adipose tissue inflammation occurs in a sex-dependent manner. Here we have employed the clinically relevant NIAAA model of chronic-binge alcohol consumption to investigate this sexual dimorphism. We report that female mice have greater liver injury than male mice despite lower alcohol consumption. Chronic-binge alcohol induces adipose tissue inflammation *in vivo* in female mice, which is illustrated by increased expression of TNFα, IL-6, and CCL2, compared to only IL-6 induction in male adipose tissue. Further, macrophage activation markers such as CD68 as well as the pro-inflammatory activation markers CD11b and CD11c were higher in female adipose tissue. Interestingly, alcohol induced expression of TLR2, 3, 4, and 9 in female but not male adipose tissue, without affecting the TLR adaptor, MyD88. Higher trends of serum endotoxin in female mice may likely contribute to adipose tissue inflammation. *In vitro* chronic alcohol-mediated sensitization of macrophages to endotoxin is independent of sex. In summary, we demonstrate for the first time that there is a sexual dimorphism in alcohol-induced adipose tissue inflammation and female mice exhibit a higher degree of inflammation than male mice.

## Introduction

Alcoholic liver disease (ALD) represents a spectrum of liver injury, starting with steatosis and progressing towards alcoholic hepatitis, fibrosis, cirrhosis, and hepatocellular carcinoma (HCC). ALD develops as a result of chronic, sustained drinking and is driven by both metabolic and immune insults [1, 2]. Chronic alcohol exposure causes triglyceride accumulation in hepatocytes and promotes liver inflammation via endotoxin derived from the gut [3–8].

In humans it is well established that women are more susceptible than men to develop ALD, despite consuming lower amounts of alcohol. For any amount of alcohol intake, women have a greater risk of developing ALD and progressing to cirrhosis than men [1, 9–12]. Several factors have been proposed that may contribute to this phenomenon. First, women have a lower first pass metabolism (FPM) than men due to lower gastric alcohol dehydrogenase activity [13, 14]. Second, women have a lower volume of distribution of alcohol than men due to lower total body water content and higher body fat content [13, 15]. Third, estrogen can greatly influence alcohol-induced liver injury. Estrogens sensitize Kupffer cells to endotoxin injury [16, 17]. Rodents exhibit this sexual dimorphism as well [18–20]. Ovariectomized rats are protected from early alcohol-induced liver injury and supplementing with exogenous estrogen reverses this effect [21].

In addition to the direct metabolic and immune impacts on the liver, the influence of alcohol on adipose tissue inflammation is being studied. In both mice and rats chronic alcohol consumption induces expression of pro-inflammatory cytokines and chemokines in white adipose tissue [22–28]. In humans, the production of cytokines in adipose tissue is correlated with acute alcoholic hepatitis (AAH) score in a cohort of patients with ALD [29]. Cytokine production in adipose tissue decreases in patients with mild liver lesions one week after cessation of drinking [30]. Previous data has shown that chronic alcohol consumption decreases the production of adiponectin, a major adipokine, in rodent models [31–33]. Infusing alcohol-fed mice with full-length recombinant adiponectin reverses liver injury [33]. These studies demonstrate the impact of alcohol consumption on adipose tissue and the link between the adipose tissue and the liver. However, differences in body composition, with regards to women having higher body fat content, are not considered [15]. Therefore it remains to be determined whether sex-dependent consequences of alcohol consumption affect adipose tissue inflammation.

The aim of this study was to determine if adipose tissue inflammation also exhibits a sexually dimorphic response to alcohol consumption. Here we show that female mice have higher liver injury compared to male mice in a model of alcoholic liver disease. Using the clinically relevant NIAAA model of chronic-binge alcohol feeding we establish that alcohol-induced adipose tissue inflammation occurs to a higher degree in female mice, compared to male mice.

## Materials and Methods

### Animals and experimental models

Ten-week old male and female C57BL/6J mice were purchased from Jackson Laboratories (Bar Harbor, ME). Mice were subjected to the NIAAA model, which recapitulates the chronic-binge drinking patterns of alcoholic hepatitis patients, as described earlier [34]. Briefly, a total of 18 mice per sex were divided into two groups (n = 9 per group). One group was fed a 5% ethanol (v/v) Lieber-DeCarli diet (Bio-Serv, Flemington, NJ.) for 10 days, following a one-week ramp up period. On the eleventh day, mice received an ethanol gavage (5 g/kg body weight, 31.5% ethanol) and were sacrificed nine hours later. The other group was fed an isocaloric control diet during the feeding and a maltose dextrin (Bio-Serv, Flemington, NJ.) gavage was administered nine hours before sacrifice. Blood was collected for serum isolation. Perigonadal adipose tissue was excised and fixed in 10% buffered formalin or snap frozen. Livers were excised and snap frozen, preserved in RNA*later* RNA Stabilizing Reagent (Qiagen GmbH, Hilden, Germany), fixed in 10% buffered formalin for histological analysis, or frozen in O.C.T. Compound (Tissue-Tek, Sakura Finetek USA, Inc., Torrance, CA).

### Ethics statement

All animals received proper care in accordance with the *Guide for the Care and Use of Laboratory Animals* from the National Institutes of Health. The protocol was approved by the Institutional Animal Care and Use Committee of the University of Massachusetts Medical School (protocol number A-2393-15). Animals were euthanized by carbon dioxide asphyxiation followed by cervical dislocation.

### Serum analysis

Blood alcohol content was measured in serum using the Alcohol Reagent and the AM1 Alcohol Analyzer (Analox Instruments Ltd., London, UK). Serum alanine aminotransferase levels were measured using the Liquid ALT reagent set (Pointe Scientific Inc., Canton, MI). Serum adiponectin levels were measured using the Mouse Adiponectin/Acrp30 Quantikine ELISA kit (R&D systems, Minneapolis, MN). Serum endotoxin was measured using the Limulus Amebocyte Lysate (LAL) QCL-1000 kit (Lonza Walkersville, Inc., Walkersville, MD).

### Liver triglycerides

Liver triglycerides were extracted and quantified as follows: liver tissue was homogenized in a 5% NP-40 solution. Samples were heated to 95°C for five minutes, cooled on ice, and then subsequently heated to 95°C for another five minute incubation. Samples were spun at 14,000 rpm in a room temperature centrifuge, and supernatants were used to quantify triglycerides with the L-Type TG M kit (Wako Diagnostics, Wako Life Sciences Inc., Mountain View, CA).

### Histological analysis

Sections of formalin-fixed adipose tissue samples were embedded and stained with hematoxylin and eosin and were analyzed for histological features. Frozen liver sections were stained with Oil Red O. Images were captured using an Olympus BX51 microscope (Olympus, Waltham, MA) and NIS-Elements Advance Research software (Nikon Instruments Inc., Melville, NY). Oil Red O quantitation was performed using Fiji software [35, 36].

### RNA extraction, cDNA synthesis, and qPCR analysis

Total RNA was extracted from RNA*later* preserved livers and flash frozen adipose tissue using the RNeasy Mini Kit (Qiagen GmbH, Hilden, Germany), according to manufacturer’s instructions. Adipose tissue was homogenized in QIAzol (Qiagen GmbH, Hilden, Germany) and subjected to a chloroform extraction before proceeding with RNA isolation. RNA concentration was measured with a NanoDrop 2000 (ThermoScientific, Wilmington, DE). Adipose tissue cDNA was synthesized using the Reverse Transcription System (Promega, Madison, WI) and liver cDNA was synthesized using the iScript Reverse Transcription Supermix for RT-qPCR (Biorad Laboratories, Hercules, CA). mRNA transcript levels were quantified using iTAQ Universal SYBR Green Supermix and CFX Connect Real-Time PCR Detection System (Biorad Laboratories, Hercules, CA) and normalized to 18s ribosomal RNA. Primer sequences are listed in Table 1. Ly6C expression was quantified using QuantiTect Primer assay #QT00247604 (Qiagen GmbH, Hilden, Germany).

**Table 1:** List of primer sequences, 5'-3'.

Gene	Forward	Reverse
18s	GTAACCCGTTGAACCCCATT	CCATCCAATCGGTAGTAGCG
CCL2	CAGGTCCCTGTCATGCTTCT	TCTGGACCCATTCCTTCTTG
CCR2	GTGTACATAGCAACAAGCCTCAAAG	CCCCCACATAGGGATCATGA
CD11b	ATGGACGCTGATGGCAATACC	TCCCCATTCACGTCTCCCA
CD11c	CTGGATAGCCTTTCTTCTGCTG	GCACACTGTGTCCGAACTCA
CD68	CCCACAGGCAGCACAGTGGAC	TCCACAGCAGAAGCTTTGGCCC
F4/80	TGCATCTAGCAATGGACAGC	GCCTTCTGGATCCATTTGAA
IL-6	ACAACCACGGCCTTCCCTACTT	CACGATTTCCCAGAGAACATGTG
MyD88	AGAACAGACAGACTATCGGCT	CGGCGACACCTTTTCTCAAT
TLR2	ACAATAGAGGGAGACGCCTTT	AGTGTCTGGTAAGGATTTCCCAT
TLR3	AATCCTTGCGTTGCGAAGTG	ACCCCGGGGAGAACTCTTTA
TLR4	GCCTTTCAGGGAATTAAGCTCC	AGATCAACCGATGGACGTGTAA
TLR9	TCCCAACATGGTTCTCCGTC	GGTGGTGGATACGGTTGGAG
TNFα	GAAGTTCCCAAATGGCCTCC	GTGAGGGTCTGGGCCATAGA

18s: 18s ribosomal RNA, CCL2: chemokine (C-C motif) ligand 2, CCR2: chemokine (C-C motif) receptor 2, CD11b: Itgam: integrin alpha M, CD11c: Itgax: integrin alpha x, CD68: cd68 antigen, F4/80: Emr1: EGF-like module containing, mucin-like, hormone receptor-like sequence 1, IL-6: interleukin 6, MyD88: myeloid differentiation primary response gene 88, TLR2: toll-like receptor 2, TLR3: toll-like receptor 3, TLR4: toll-like receptor 4, TLR9: toll-like receptor 9, TNFα: tumor necrosis factor.

### Bone marrow-derived macrophages

Bone marrow was harvested from the femurs of 10-week old male and female C57BL/6J mice. Bone marrow cells were plated in RMPI 1640 (Thermo Fisher Scientific, Waltham, MA) supplemented with 10% Fetal Bovine Serum (Gemini Bio-Products, West Sacramento, CA), 1% Penicillin Streptomycin (Thermo Fisher Scientific, Waltham, MA), and 1% L-glutamine 200mM (Thermo Fisher Scientific, Waltham, MA) (“complete RPMI”) onto non-tissue culture treated 10 cm plates in the presence of 50 ng/mL Recombinant Murine Macrophage Colony Stimulating Factor (M-CSF) (Peprotech, Rocky Hill, NJ) to induce differentiation. On day four of differentiation, cells were supplemented with fresh complete RMPI with 50 ng/mL M-CSF. On day 6, cells were harvested in 1mM EDTA in PBS and seeded in 6-well plates at a density of one million cells per well, in the presence of 50 ng/mL M-CSF and allowed to adhere overnight. After adherence, cells were cultured in the presence of 5 ng/mL M-CSF. To mimic chronic alcohol conditions, BMDMs were cultured in 25 mM ethanol in humidified chambers for 5 days as described previously by our group [37]. Cells were stimulated with 100 ng/mL LPS (Sigma-Aldrich Corp., St. Louis, MO) for 18 hours, at which point supernatants were collected and BMDMs were counted for ELISA analysis. The BD OptEIA Mouse TNFα (Mono/Mono) ELISA set (BD Biosciences, San Diego, CA) was used to measure TNFα protein in cell supernatants. TNFα protein concentration was normalized to cell number.

### Statistical analysis

Data are represented as mean ± SEM. All statistical analysis was performed using Graphpad Prism 6.0. Student’s t-test was used to determine the difference between two groups; Two-way ANOVA was used to determine the difference between four groups.

## Results

### The NIAAA ALD model induces greater liver injury in female mice despite lower alcohol consumption

**Fig 1:**
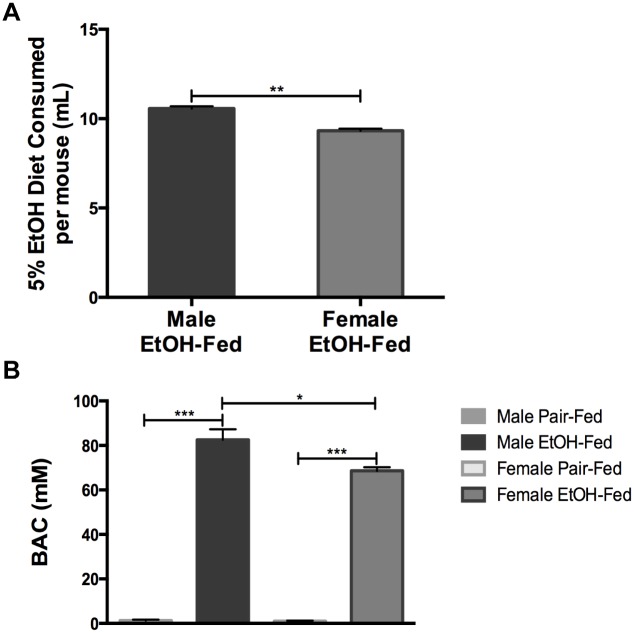
Male mice consume higher amounts of alcohol. Male and female mice were subjected to the NIAAA model. (A) Average daily consumption of the 5% Lieber-DeCarli diet per mouse. (B) Serum blood alcohol content (BAC) at the time of sacrifice. * p<0.05, ** p<0.01, *** p<0.001. Data are represented as mean ± SEM. EtOH-fed: Alcohol-fed.

**Fig 2:**
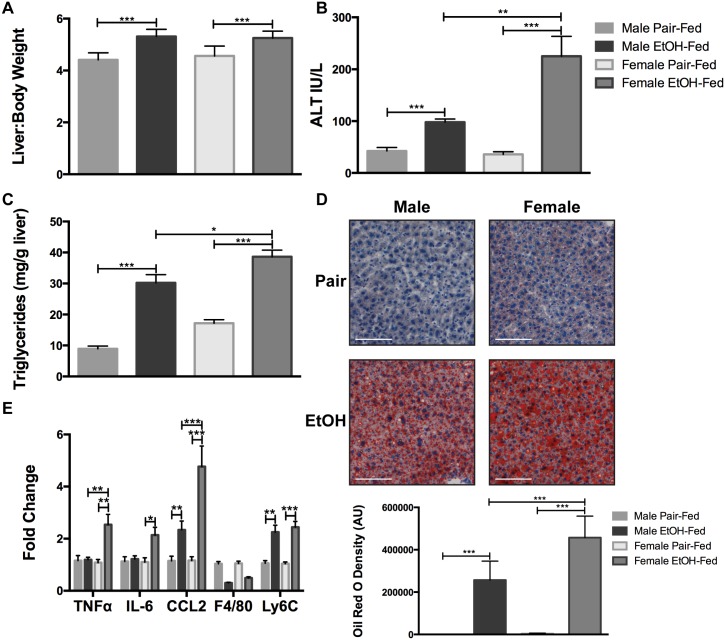
Female mice have more severe liver injury than male mice. (A) Liver-to-body weight ratio. (B) Serum alanine aminotransferase (ALT) levels at the time of sacrifice. (C) Liver triglyceride content. (D) Representative Oil Red O staining of liver sections. Scale bar is set to 100 μm and magnification is at 100x. Quantitation was done with Fiji. (E) Liver mRNA levels of TNFα, IL-6, CCL2, F4/80, and Ly6C were quantified by qPCR. * p<0.05, ** p<0.01, *** p<0.001. Data are represented as mean ± SEM.

In order to compare the extent of liver injury between male and female mice, ten-week old male and female C57BL/6J mice were subjected to the NIAAA model of chronic-binge alcohol feeding. Over the course of the experiment, male mice consumed higher amounts of the 5% alcohol diet than female mice (Fig 1A). In support of higher alcohol consumption, male mice had a higher serum blood alcohol content (BAC) than female mice at the end of the study (Fig 1B). Regardless of the volume of alcohol that was consumed, male and female mice had similar increases in liver-to-body weight ratios (Fig 2A). Furthermore, despite consuming lower amounts of alcohol, female mice had higher serum alanine aminotransferase (ALT) levels than their male counterparts (~2.3-fold) (Fig 2B). Female mice also had elevated liver triglyceride content compared to male mice (Fig 2C), which was confirmed by Oil Red O staining (Fig 2D). Gene expression analysis of liver pro-inflammatory cytokines showed a significant upregulation of CCL2 mRNA in both male and female mice, however, female mice had greater CCL2 expression than male mice. IL-6 and TNFα expression were increased in female mice. The expression of the macrophage marker F4/80 was decreased in both male and female mice, but the change was not significant. Interestingly, the expression of the monocyte marker Ly6C was increased in male and female mice (Fig 2E). Together, these data show that female mice exhibit a higher degree of alcohol-induced liver injury than male mice.

### Chronic-binge alcohol feeding induces greater adipose tissue inflammation in female mice than in male mice

**Fig 3:**
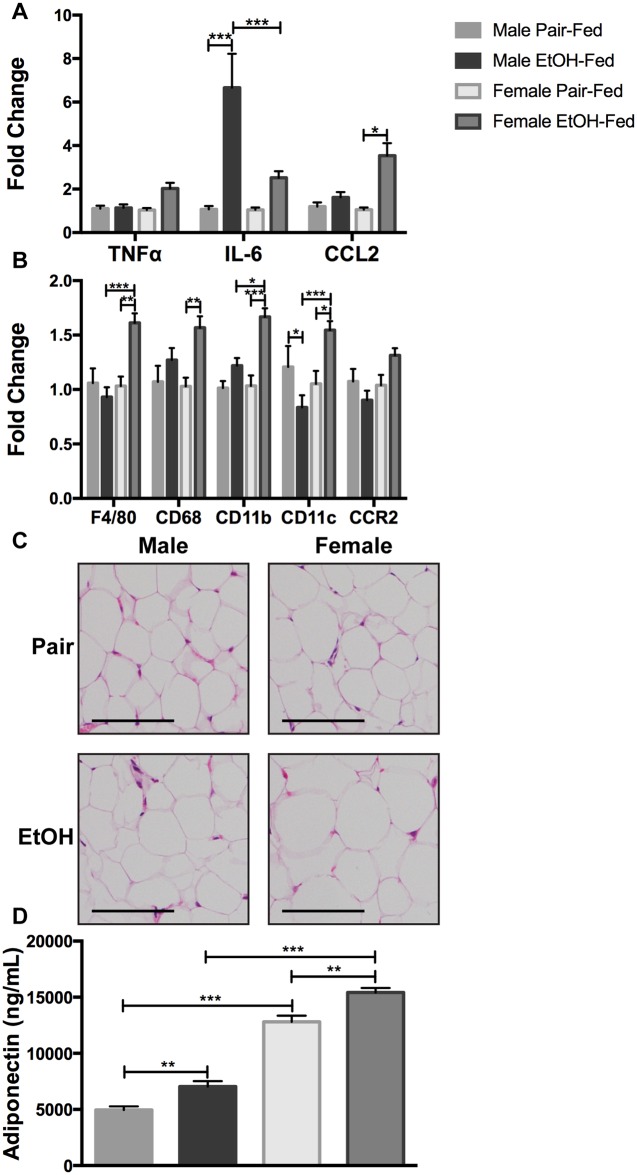
Female mice have a higher degree of adipose tissue inflammation. Periogonadal adipose tissue mRNA levels of (A) TNFα, IL-6, and CCL2 and (B) F4/80, CD68, CD11b, CD11c, and CCR2 were quantified by qPCR. (C) H&E staining of adipose tissue. Scale bar is set to 100 μm and magnification is at 100x. (D) Serum adiponectin levels were measured by ELISA.* p<0.05, ** p<0.01, *** p<0.001. Data are represented as mean ± SEM.

Considering sex-dependent differences in human body fat composition, we wanted to determine if adipose tissue also exhibits a sexually dimorphic response to chronic-binge alcohol exposure. Perigonadal adipose tissue was collected and analyzed for expression of pro-inflammatory cytokines, chemokines, and immune cell markers. Pro-inflammatory cytokines and chemokines exhibited a sexually dimorphic profile, wherein there was a highly significant increase in expression of IL-6 mRNA in male mice and a trend of increase noted in female mice. On the other hand, CCL2 mRNA was increased only in female mice. TNFα mRNA levels were unchanged in male adipose tissue but female adipose tissue showed a trend towards increased expression (Fig 3A). Alcohol consumption increased the expression of the macrophage marker F4/80 and the activation markers CD68, CD11b, and CD11c in female, but not male mice, without any effect on CCR2 (Fig 3B). Histological analysis showed no overt differences in adipose tissue morphology between the sexes (Fig 3C). Unexpectedly, chronic-binge alcohol did not reduce serum adiponectin levels. In this chronic-binge model, alcohol increased adiponectin production in both male and female mice, with higher levels in female mice (Fig 3D). Overall, these results show that chronic-binge alcohol induces adipose tissue inflammation in female mice concomitant to higher liver injury.

### Chronic-binge alcohol feeding increases circulating endotoxin and innate immune activation markers in female mice

**Fig 4:**
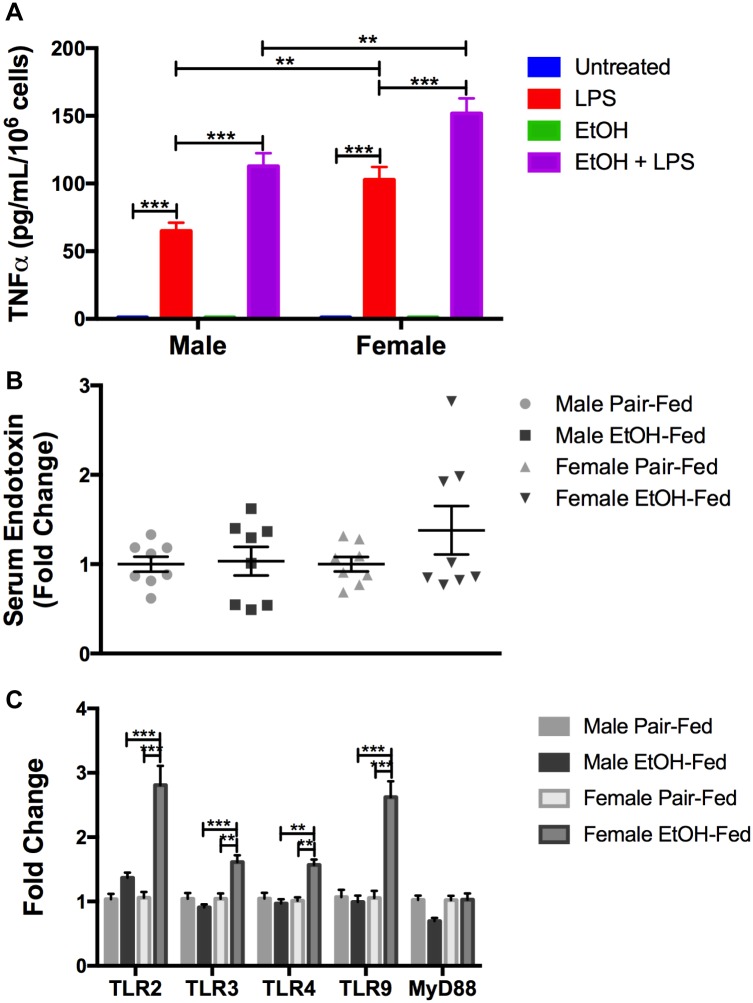
Increased adipose tissue inflammation is due to innate immune cells. (A). BMDMs were cultured in the presence of 25mM ethanol for 5 days and then stimulated with 100ng/mL LPS for 18 hours. TNFα was measured in the cell supernatants by ELISA. (B) Serum endotoxin levels at time of sacrifice. (C) Adipose mRNA levels of TLR2, TLR3, TLR4, TLR9, and MyD88 were quantified by qPCR. * p<0.05, ** p<0.01, *** p<0.001. Data are represented as mean ± SEM.

Alcohol sensitizes macrophages to endotoxin/lipopolysaccharide (LPS) stimulation, due to alcohol-induced oxidative stress [38]. To determine whether macrophages are sensitized to alcohol in a sexually dimorphic manner, we exposed bone marrow derived macrophages (BMDMs) from male and female mice *in vitro* to chronic alcohol and analyzed cytokine expression to a subsequent challenge by LPS. Female derived BMDMs produced more TNFα than male derived BMDMs in response to LPS, in the presence or absence of alcohol (Fig 4A). However, alcohol sensitized male and female BMDMs equally significantly; alcohol increased LPS-induced TNFα production to 1.7 fold in male BMDM and 1.5 fold in female BMDMs, compared to LPS alone (Fig 4A).

Since alcohol-induced macrophage sensitization to LPS stimulation did not differ between male and female macrophages, we wanted to determine if there were sex-dependent differences in circulating LPS, the expression of TLRs that are important in its recognition, or both [39, 40]. Previous data in models of ALD show high circulating endotoxin in female rodents, compared to their male counterparts [18, 19]. We show that serum endotoxin levels are unchanged in alcohol-fed male mice compared to pair-fed male mice, whereas a trend of increase in alcohol-fed female mice (approximately 1.4 fold) compared to pair-fed female mice was noted (Fig 4B). Further, female adipose tissue, but not male adipose tissue, showed an increase in several TLR genes: TLR2, TLR3, TLR4, and TLR9, indicating either an upregulation of these genes in innate immune cells resident in the adipose tissue or an increased number of TLR-expressing immune cells in alcohol-fed female mice (Fig 4C). Expression of another common adaptor gene MyD88 was unchanged in both sexes. This data shows that while alcohol does not sensitize macrophages in a sex-dependent manner, circulating endotoxin and increased expression of TLRs in female mice could be potential mechanisms contributing to the sexual dimorphism in alcohol-induced adipose tissue inflammation.

## Discussion

The sexual dimorphism in human alcoholic liver disease is well known; women are more likely than men to develop ALD, despite consuming less alcohol [1, 9–12]. Human studies and rodent models indicate differences in first-pass metabolism, body water content, and hormones may contribute to the sexual dimorphism in ALD. [13–17]. Whether sexual differences play an important part in alcohol mediated adipose tissue inflammation and its impact on liver disease is not yet considered. Our studies show that chronic alcohol consumption causes adipose tissue inflammation in a sex-dependent manner.

Here we provide an analysis of the sexual dimorphism in adipose inflammation that occurs in mice when subjected to the NIAAA model of chronic-binge alcohol exposure. This model employs a single binge of alcohol delivered at the end of the study, which mimics a binge event common in alcoholic hepatitis patients [34]. Our studies show that male mice consumed higher volumes of the 5% alcohol diet and had a higher BAC than their female counterparts on an *ad libitum* diet. Despite this, female mice had greater liver injury, as shown by elevated serum ALT and high liver triglycerides, similar to previous studies from Ki and colleagues [20]. From these studies, it is apparent that increased serum ALT and triglycerides occur despite lower alcohol consumption and BAC in female mice.

The production of pro-inflammatory cytokines and chemokines in the liver has been reported in male and female mice subjected to the chronic-binge alcohol model [20, 41, 42]. Here we show a direct comparison of cytokine, chemokine, and monocyte/macrophage marker mRNA expression between male and female mice. We report an increase in TNFα, IL-6, and CCL2 mRNA in alcohol-fed female mice whereas alcohol-fed male mice exhibit an increase in CCL2 mRNA. Furthermore, alcohol-fed female mice have greater CCL2 expression than alcohol-fed male mice in the liver. Previous studies using male mice have shown minimal or lack of expression of TNFα, IL-6, and MCP-1/CCL2 mRNA in the liver [43, 44]. Expression of the macrophage marker F4/80 is decreased in both male and female livers. This decrease has been reported in other chronic alcohol models and is likely a result of the resident Kupffer cells undergoing apoptosis [41, 45]. Ly6C mRNA, a marker of pro-inflammatory monocytes, is increased in alcohol-fed male and female livers, indicating that there is infiltration of monocytes into the livers of these mice. This is in agreement with previous studies showing that chronic alcohol consumption increases the presence of Ly6C-expressing monocytes in mouse livers and that this increase is enhanced further with the administration of an ethanol binge [46]. Overall, our results show that alcohol-fed female mice exhibit higher expression of pro-inflammatory cytokines than male mice.

Adipose tissue biology has been extensively studied in the context of obesity. Low-grade adipose tissue inflammation is linked to the development of metabolic disorders, namely Type 2 diabetes. Adipose tissue inflammation is characterized by the increased presence of pro-inflammatory macrophages and the production of pro-inflammatory adipokines [47]. Similar to studies on obesity, studies in ALD models have shown that chronic alcohol consumption can induce pro-inflammatory responses in the adipose tissue. Female mice on an *ad libitum* alcohol diet produce cytokines in the adipose tissue and exhibit increased expression of the pro-inflammatory macrophage marker CD11c [23]. In other chronic ALD models, male rats fed alcohol *ad libitum* for four weeks exhibit increased TNFα, IL-6, and CCL2 production in adipose tissue [24, 26]. Male mice fed alcohol for four to eight weeks also reveal increases in adipose tissue cytokine production [22, 25]. Our data show higher expression of TNFα, IL-6, and CCL2 in female adipose tissue, whereas increased IL-6 but not TNFα or CCL2 was observed in male adipose tissue using the NIAAA model of chronic-binge alcohol feeding. It is likely that pro-inflammatory cytokine induction in male adipose tissue may further increase with a prolonged (4–8 weeks) alcohol-feeding regimen. Although previously published studies provide evidence for chronic alcohol consumption-mediated adipose tissue inflammation, they have not explored whether sex-dependent differences exist in alcohol-induced adipose tissue inflammation.

We report for the first time that alcohol-induced adipose tissue inflammation occurs in a sex-dependent manner. Male and female adipose tissues have a distinctly different cytokine signature in response to the chronic-binge alcohol model: male mice express high IL-6 mRNA, whereas female mice express elevated CCL2 mRNA. The role of IL-6 in adipose tissue function is controversial; evidence exists in support of both its pro- and anti-inflammatory functions [47, 48]. It is likely that the increase in IL-6 expression in male adipose tissue exerts an anti-inflammatory response. On the other hand, increased CCL2 in female adipose tissue could be indicative of a pro-inflammatory state, which is consistent with obesity-related adipose tissue inflammation [47]. Increased CCL2 expression in male adipose tissue, but not female, correlates to a higher degree of inflammation in high fat diet models, in which male mice have more severe metabolic dysfunction [49]. Interestingly, we observe an opposite paradigm; females exhibit higher CCL2 and are more susceptible than males to liver injury. In mouse models of obesity, there is increased numbers of pro-inflammatory macrophages in the adipose tissue [50–53]. In our data, female mice show an increase in the expression of a number of macrophage markers (F4/80, CD68, CD11b, and CD11c). This indicates either an increase in the amount of macrophages within the adipose tissue or increased expression of these markers, suggesting that resident adipose macrophages likely acquire a pro-inflammatory phenotype. Interestingly, H&E staining of adipose tissue sections show that adipocyte morphology remains unchanged between the sexes and pair-and alcohol-fed groups. There is an absence of crown-like structures, which indicates that macrophage activation is occurring without a significant change in macrophage numbers by alcohol [47].

Consistent with previous results, female mice had higher serum adiponectin in control animals than male mice [32]. In contrast to previous rodent studies using long-term chronic alcohol consumption models, the NIAAA model employed here did not show reduced serum adiponectin levels [28, 33, 38]. Serum adiponectin was increased in both male and female mice, likely due to a compensatory mechanism. Studies in ALD patients report increased serum adiponectin levels when compared to healthy controls or hepatitis C patients. Serum adiponectin is positively correlated with severity of liver injury [54–56]. One possible explanation for the discrepancy between the mouse models could be the difference in drinking patterns because the NIAAA model includes a binge, whereas other models do not. Our model is reflective of the drinking patterns in alcoholic hepatitis patients, suggesting that future studies on serum adiponectin alcohol models employing a binge are needed [34].

We sought to identify the underlying mechanism that contributes to the sexual dimorphism in adipose tissue inflammation. Chronic alcohol exposure increases the sensitivity of macrophages to endotoxin-mediated TNFα production and estrogen also sensitizes liver macrophages to endotoxin injury [16, 38]. Here we investigated the effect of chronic alcohol exposure on male- and female-derived BMDMs. BMDMs exposed to chronic alcohol conditions *in vitro* increased LPS-induced TNFα production; however the extent of increase due to alcohol exposure was similar between both male- and female-derived BMDMs. It is likely that the increased expression of macrophage markers in the adipose tissue is regulated to some extent by the infiltration of bone marrow-derived immune cells during alcohol exposure. Furthermore, circulating endotoxin showed higher trends in alcohol-fed female mice compared to male counterparts. The increased endotoxin could contribute to adipose inflammation in female mice. Moreover, the expression of several TLR genes was increased in the adipose tissue of female, but not male mice. This indicates either an increased presence of TLR-expressing cells or an upregulation of these genes in resident macrophages in females but not in males. The exact mechanism of sex-dependent upregulation of TLRs during alcohol exposure remains to be further explored. Overall our data provides some insights regarding how the propensity of increased circulating endotoxin in alcohol-fed female mice could lead to greater adipose tissue inflammation, dependent on mechanisms that are associated with increased TLR expression and/or macrophage activation.

This work highlights the role of adipose tissue inflammation within the context of sexual dimorphism that is observed in human ALD. In conjunction with our studies, it will be imperative that all forthcoming alcohol studies carefully consider biological sex as an experimental variable. This will be important particularly in interpreting current and future studies that attempt to target adipose tissue as a means of resolving alcohol-induced liver injury. It is crucial that therapeutic strategies for alcoholic hepatitis patients are designed considering appropriate targets and the biologic sex of the patient.
